# Screening and Functional Pathway Analysis of Pulmonary Genes Associated with Suppression of Allergic Airway Inflammation by Adipose Stem Cell-Derived Extracellular Vesicles

**DOI:** 10.1155/2020/5684250

**Published:** 2020-06-27

**Authors:** Sung-Dong Kim, Shin Ae Kang, Yong-Wan Kim, Hak Sun Yu, Kyu-Sup Cho, Hwan-Jung Roh

**Affiliations:** ^1^Department of Otorhinolaryngology and Biomedical Research Institute, Pusan National University Hospital, Busan, Republic of Korea; ^2^Department of Parasitology and Tropical Medicine, Pusan National University School of Medicine, Yangsan, Republic of Korea; ^3^Department of Otorhinolaryngology, Inje University Haeundae Paik Hospital, Republic of Korea; ^4^Department of Otorhinolaryngology and Research Institute for Convergence of Biomedical Science and Technology, Pusan National University Yangsan Hospital, Yangsan, Republic of Korea

## Abstract

**Background:**

Although mesenchymal stem cell- (MSC-) derived extracellular vesicles (EVs) are as effective as MSCs in the suppression of allergic airway inflammation, few studies have explored the molecular mechanisms of MSC-derived EVs in allergic airway diseases. The objective of this study was to evaluate differentially expressed genes (DEGs) in the lung associated with the suppression of allergic airway inflammation using adipose stem cell- (ASC-) derived EVs.

**Methods:**

C57BL/6 mice were sensitized to ovalbumin (OVA) by intraperitoneal injection and challenged intranasally with OVA. To evaluate the effect of ASC-derived EVs on allergic airway inflammation, 10 *μ*g/50 *μ*L of EVs were administered intranasally prior to OVA challenge. Lung tissues were removed and DEGs were compared pairwise among the three groups. DEG profiles and hierarchical clustering of the identified genes were analyzed to evaluate changes in gene expression. Real-time PCR was performed to determine the expression levels of genes upregulated after treatment with ASC-derived EVs. Enrichment analysis based on the Gene Ontology (GO) database and Kyoto Encyclopedia of Genes and Genomes (KEGG) pathway analysis were also performed to further identify the function of DEGs.

**Results:**

Expression of paraoxonase 1 (PON1), brain-expressed X-linked 2 (Bex2), insulin-like growth factor binding protein 6 (Igfbp6), formyl peptide receptor 1 (Fpr1), and secretoglobin family 1C member 1 (Scgb1c1) was significantly increased in asthmatic mice following treatment with ASC-derived EVs. GO enrichment and KEGG pathway analysis showed that these genes were strongly associated with immune system processes and their regulation, cellular processes, single-organism processes, and biological regulation.

**Conclusion:**

These results suggest that the DEGs identified in this study (PON1, Bex2, Igfbp6, Fpr1, and Scgb1c1) may be involved in the amelioration of allergic airway inflammation by ASC-derived EVs.

## 1. Introduction

Asthma is a chronic inflammatory airway disease involving multiple cellular components; its key features are airway hyperresponsiveness (AHR), persistent airway inflammation, and airway remodeling [1]. Excessive activation of Th2 cells by insufficient suppression of regulatory T cells (Tregs) plays an important role in the pathogenesis of allergic airway inflammation [2–4]. Recently, airway remodeling was reported to be important in pathological pathways of asthma characterized by irreversible AHR and airway obstruction [5].

Mesenchymal stem cells (MSCs) modulate immune responses and inflammation [6]. Several studies have shown that MSCs, including those derived from adipose tissue (ASCs), can improve allergic airway inflammation in asthmatic mice [7–9]. Although the immune suppression mechanism of MSCs in allergic airway diseases is not completely understood, it has been demonstrated to be strongly related to Treg upregulation and increases in soluble factors such as prostaglandin E2 (PGE2), transforming growth factor-*β* (TGF-*β*), and interleukin- (IL-) 10 [10–13]. MSCs have also been shown to modulate recognition of antigen-presenting cells that mediate cellular immune responses, including dendritic cells, macrophages, and B cells [14, 15].

Several recent studies have shown that T cell activation and proliferation are suppressed by the MSC culture supernatant (MSC sup) [16, 17]. Accumulating evidence shows that administration of MSC sup or extracellular vesicles (EVs) released by MSCs is as effective as that of MSCs in suppressing allergic airway inflammation [18–20]. MSC-derived EVs were found to upregulate IL-10 and TGF-*β*1 from peripheral blood mononuclear cells of asthmatic patients, thereby promoting the proliferation and immune suppression capacity of Tregs [21]. Furthermore, ASC-derived EVs ameliorated Th2-mediated inflammation induced by the *Aspergillus* protease antigen through the activation of dendritic cells and induction of M2 macrophage polarization [22]. Although a recent study showed that MSC-derived EVs prevented group 2 innate lymphoid cell-dominant allergic airway inflammation through miR-146a-5p [23], the molecular mechanisms of MSC-derived EVs in allergic airway inflammation remain to be elucidated, and the genes involved in these mechanisms have not been definitively identified.

In this study, we isolated EVs secreted by ASCs and performed microarray gene expression analysis in asthmatic mice treated with ASC-derived EVs. We also examined differentially expressed genes (DEGs) associated with the suppression of allergic airway inflammation by ASC-derived EVs.

## 2. Materials and Methods

### 2.1. Animals

Six-week-old female C57BL/6 mice were purchased from Samtako Co. (Osan, Republic of Korea) and bred in animal facilities without specific pathogens during experiments. The animal study protocol was approved by the Institutional Animal Care and Use Committee of the Pusan National University School of Medicine (Approval No. PNU-2016-1109).

### 2.2. EV Extraction and Characterization

As in our previous study [11, 24, 25], adipose tissue was obtained from the abdominal fat of C57BL/6 mice. ASCs were cultured at 37°C with 5% CO_2_ in *α*-modified Eagle's medium (*α*-MEM) containing 10% fetal bovine serum (FBS) until 1 × 10^6^ cells/cm^2^ were obtained. EVs were isolated from ASC sup as previously described [26]. The supernatant was filtered through a 0.45 *μ*m vacuum filter. The filtrate was concentrated using QuixStand (GE Healthcare, Little Chalfont, UK) and then filtered through a 0.22 *μ*m bottle top filter (Sigma-Aldrich, St. Louis, MO). The filtrates were pelleted by ultracentrifugation in a 45 Ti rotor (Beckman Coulter, Fullerton, CA) at 100,000 × *g* for 2 h at 4°C. The final pellets were resuspended in phosphate-buffered saline (PBS) and stored at -80°C. We placed the EVs in PBS on 300-mesh copper grids and stained them with 2% uranyl acetate. Images were obtained using a JEM-1011 electron microscope (JEOL, Tokyo, Japan) operated at an acceleration voltage of 100 kV [27, 28]. EV markers including CD81 and CD40 were analyzed by western blotting with primary antibodies, anti-CD81 (1 : 1000, Abcam, Cambridge, MA), and anti-CD40 (1 : 1000, Abcam) as previously described [22].

### 2.3. Mouse Model of Allergic Airway Inflammation

**Figure 1:**
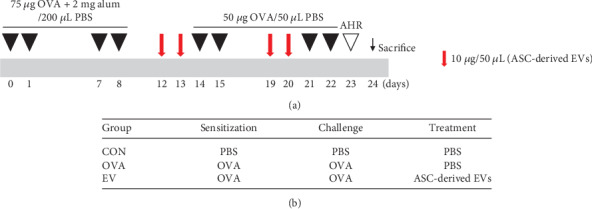
Experimental protocol of this study. (a) Mice were sensitized on days 0, 1, 7, and 8 by intraperitoneal injection of ovalbumin (OVA) and challenged intranasally on days 14, 15, 21, and 22 with OVA. Adipose stem cell- (ASC-) derived extracellular vesicles (EVs) (10 *μ*g/50 *μ*L) were injected intranasally on days 12, 13, 19, and 20. (b) Mice were divided into three groups according to sensitization, challenge, and treatment.

A mouse model of allergic airway inflammation was induced as previously reported with minor modifications [24, 25]. The mice were sensitized by intraperitoneal injection of 75 *μ*g of OVA (Sigma-Aldrich, St. Louis, MO, USA) with 2 mg of aluminum hydroxide (Sigma-Aldrich) in 200 *μ*L of PBS on days 0, 1, 7, and 8. On days 14, 15, 21, and 22, the mice were challenged intranasally with 50 *μ*g of OVA in 50 *μ*L of PBS. The mice were sacrificed on day 24 (Figure 1(a)).

### 2.4. Intranasal Administration of ASC-Derived EVs

To evaluate the effect of ASC-derived EVs, we injected 10 *μ*g/50 *μ*L of EVs intranasally on days 12, 13, 19, and 20. Mice were divided into three groups, with five mice per group: (a) the CON group was sensitized, pretreated, and challenged with PBS; (b) the OVA group was sensitized with OVA, pretreated with PBS, and then challenged with OVA; and (c) the EV group was sensitized with OVA, pretreated with ASC-derived EVs, and then challenged with OVA (Figure 1(b)).

### 2.5. Microarray Analysis of the Lung Tissue

Lung tissues were extracted and DEGs were compared pairwise among the three groups. To investigate changes in gene expression following treatment with ASC-derived EVs, microarray analyses were performed by Macrogen Inc. (Seoul, Republic of Korea), a company that specializes in this technology. The Affymetrix Whole-transcript Expression array process was performed using the GeneChip Whole Transcript PLUS Reagent Kit to extract total RNA from lung tissue according to the manufacturer's protocol. Then, cDNA was synthesized as described by the manufacturer using the GeneChip Whole Transcript (WT) Amplification Kit and sense cDNA was fragmented and biotin-labeled with terminal deoxynucleotidyl transferase (TdT) using the GeneChip WT Terminal Labeling Kit. Approximately 5.5 *μ*g of the labeled DNA target was hybridized at 45°C for 16 h to the Affymetrix GeneChip Mouse 2.0 ST Array. After washing the hybridized arrays and staining with the GeneChip Fluidics Station 450, we scanned the target using a GCS 3000 Scanner (Affymetrix) and computed the signal values using the Affymetrix GeneChip Command Console Software.

### 2.6. Gene Expression Analysis by Quantitative Real-Time Polymerase Chain Reaction (qRT-PCR)

Total RNA was extracted from lung tissues using 1 mL of QIAzol (Qiagen, Valencia, CA) following the manufacturer's protocol. We transcribed 2 *μ*g of RNA using Moloney Murine Leukemia Virus Reverse Transcriptase (Promega, Madison, WI). Paraoxonase 1 (PON1) (forward, 5′-GATTGGCACTGTGTTCCAC-3′; reverse, 5′-ATCACTGTGGTAGGCACCTT-3′), brain-expressed X-linked 2 (Bex2) (forward, 5′-GGATGTTAAAAGGGACTCCCGGTGA-3′; reverse, 5′-CGACGGCGGTTCTGACGCCACAACG-3′), insulin-like growth factor binding protein 6 (Igfbp6) (forward, 5′-GCAGCAGCTCCAGACTGA-3′; reverse, 5′-CATTGCTTCACATACAGCTCAA-3′), formyl peptide receptor 1 (Fpr1) (forward, 5′-CATGTCTCTCCTCATGAACAAG-3′; reverse, 5′-ATGAGAAGACATCCAGAACGA-3′), and secretoglobin family 1C member 1 (Scgb1c1) (forward, 5′-GGAATTCCTGCAAACACTCCT-3′; reverse, 5′-GGGCTGCTTATGTGTCCTCT-3′) RNA levels were quantified relative to the housekeeping gene glyceraldehyde 3-phosphate dehydrogenase (GAPDH) (forward, 5′-TACCCCCAATGTGTCCGTC-3′; reverse, 5′-AAGAGTGGGAGTTGCTGTTGAAG-3′), using the LightCycler 96 Real-Time PCR System (Roche, Basel, Switzerland) following the manufacturer's instructions. We used the comparative Ct (2^–ΔΔCt^) method to calculate relative gene expression levels.

### 2.7. Raw Data Preparation

We used the Affymetrix GeneChip Command Console software to extract raw data, following the Affymetrix data extraction protocol. We summarized and standardized the data using the robust multiarray average (RMA) method with the Affymetrix Expression Console software. The gene-level results were exported along with the RMA analysis, and further examined via DEG analysis.

### 2.8. Statistical Analyses

Statistical significance among the expression data was evaluated in terms of fold change. To evaluate similarity, we examined linkage and Euclidean distance among the hierarchical cluster analysis results of each DEG set. The Gene Ontology (GO) and Kyoto Encyclopedia of Genes and Genomes (KEGG) pathway databases (http://www.geneontology.org/) were used to perform gene enrichment and functional annotation analyses of significant probes. All data analyses and DEG visualization were performed using the R 3.1.2 software (R Core Team).

## 3. Results

### 3.1. Characterization of ASC-Derived EVs

Transmission electron microscopy (TEM) showed that ASC-derived EVs had lipid bilayers and were spherical in shape. Western blotting showed that ASC-derived EVs were positive for the CD81 exosome marker and CD40 microvesicle marker (data not shown).

### 3.2. Data Processing and DEG Identification

**Figure 2:**
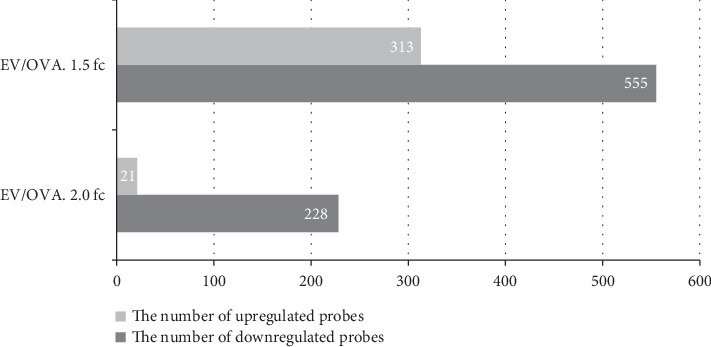
Bar plot of significant probes. We identified 868 and 249 differentially expressed genes (DEGs) with fold changes of ∣log_FC_ | ≥1.5 and ∣log_FC_ | ≥2.0 between the EV and OVA groups, respectively.

Following normalization, we analyzed DEG profiles with a false discovery rate (FDR) cut-off of FDR < 0.05 and fold change cut-offs of ∣log_FC_ | ≥1.5 and ∣log_FC_ | ≥2.0. We identified 868 DEGs with ∣log_FC_ | ≥1.5 between the EV and OVA groups, among which 313 and 555 were down- and upregulated, respectively. We identified 249 DEGs with ∣log_FC_ | ≥2.0 between the EV and OVA groups, of which 228 and 21 genes were down- and upregulated, respectively (Figure 2).

### 3.3. Hierarchical Clustering Analysis of DEGs

**Figure 3:**
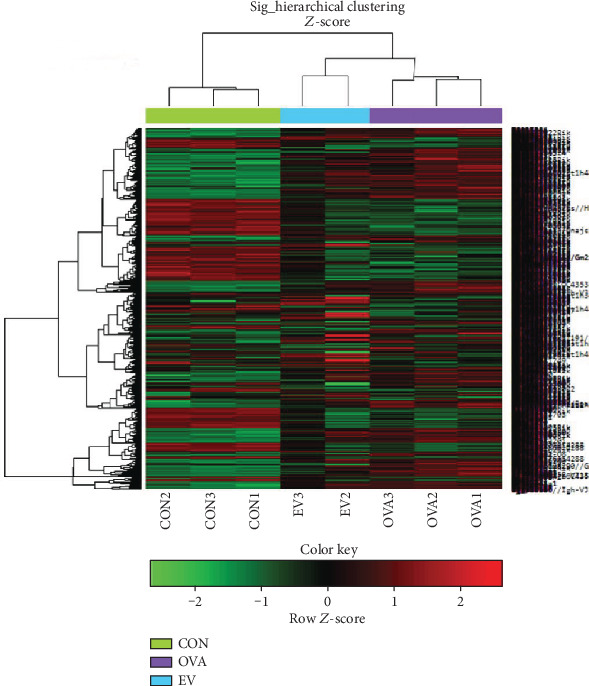
Hierarchical clustering analysis and heat map diagram. Green and red shading indicates down- and upregulated genes, respectively.

Hierarchical clustering of the identified DEGs is shown in Figure 3. Tree view and cluster analyses were performed using the Euclidean method to group and display genes with a ∣log_FC_ | ≥1.5 change in expression. Gene expression among ASC-derived EVs was compared with transcript levels among the OVA group. Upregulated and downregulated genes were easily distinguished between the two groups. Eosinophil-associated ribonuclease A family member 6 (Ear6), chemokine ligands 5, 8, and 12 (Ccl5, Ccl8, and Ccl12), tumor necrosis factor ligand superfamily member 8 (Tnfsf8), interleukin-5 receptor alpha (IL5Ra), and tumor necrosis factor receptor superfamily member 13B (Tnfrsf13b) were upregulated in the OVA group compared to the CON group, although these genes were downregulated by ASC-derived EVs (Table 1). In contrast, PON1, Bex2, Igfbp6, Fpr1, and Scgb1c1 were downregulated in OVA-induced asthmatic mice, but upregulated following treatment with ASC-derived EVs (Table 2).

### 3.4. Expression of PON1, Bex2, Igfbp6, Fpr1, and Scgb1c1

**Figure 4:**
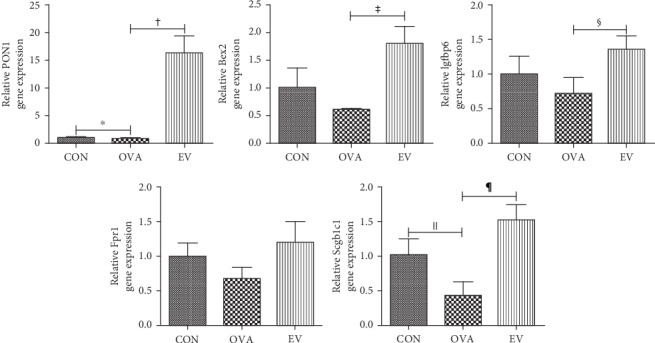
Effects of ASC-derived EVs on PON1, Bex2, Igfbp6, Fpr1, and Scgb1c1 gene expression. PON1 and Scgb1c1 gene expression was significantly decreased in the OVA group compared to the CON group. However, EV treatment markedly increased the expression of PON1, Bex2, Igfbp6, and Scgb1c1 in asthmatic mice. ^†^*p* = 0.001; ^‡^*p* = 0.003; ^§^*p* = 0.022; ^‖^*p* = 0.008; ^¶^*p* < 0.001. ASCs: adipose stem cells; Bex2: brain-expressed X-linked 2; CON: control; EV: extracellular vesicle; Fpr1: formyl peptide receptor 1; Igfbp6: insulin-like growth factor binding protein 6; OVA: ovalbumin; PON1: paraoxonase 1; Scgb1c1: secretoglobin family 1C member 1.

The gene expression levels of PON1 and Scgb1c1 were significantly decreased in the OVA group compared to the CON group (*p* = 0.001 and *p* = 0.008, respectively). However, treatment with ASC-derived EVs markedly increased the expression of PON1, Bex2, Igfbp6, and Scgb1c1 in asthmatic mice (*p* = 0.001, *p* = 0.003, *p* = 0.022, and *p* < 0.001, respectively). Although Frp1 mRNA levels increased in the EV group, there was no significant difference between the OVA and EV groups (*p* = 0.057) (Figure 4).

### 3.5. Functional Category Enrichment Analysis of DEGs

**Figure 5:**
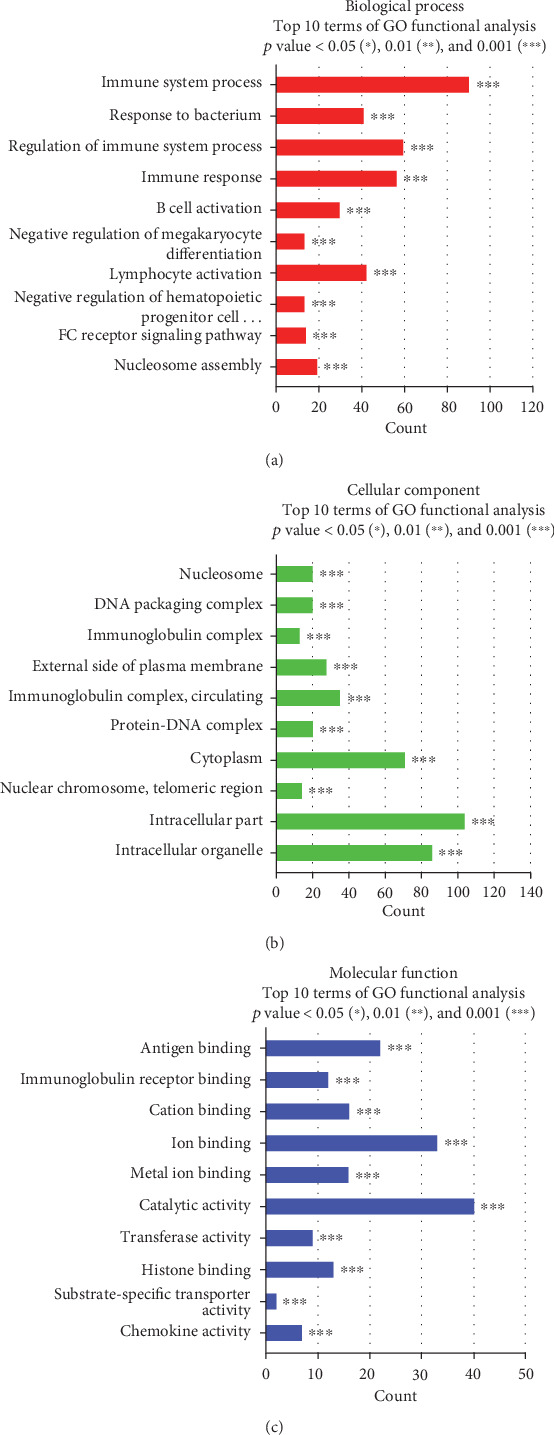
Functional category enrichment analysis of DEGs. The *y*-axis shows significantly enriched gene ontology (GO) terms, and the *x*-axis shows the counts of these terms. GO analysis included three categories: (a) biological processes, (b) cellular components, and (c) molecular function.

The GO database was used to perform enrichment analysis of DEGs to examine their association with biological processes, cellular components, and molecular functions. The 10 most highly significant terms associated with DEGs with a cut-off of FDR < 0.05 were summarized for each category. Genes that were down- and upregulated following treatment with ASC-derived EVs were strongly associated with immune system processes and their regulation (Figure 5(a)), intracellular components and intracellular organelles (Figure 5(b)), and catalytic activity and ion binding (Figure 5(c)).

**Figure 6:**
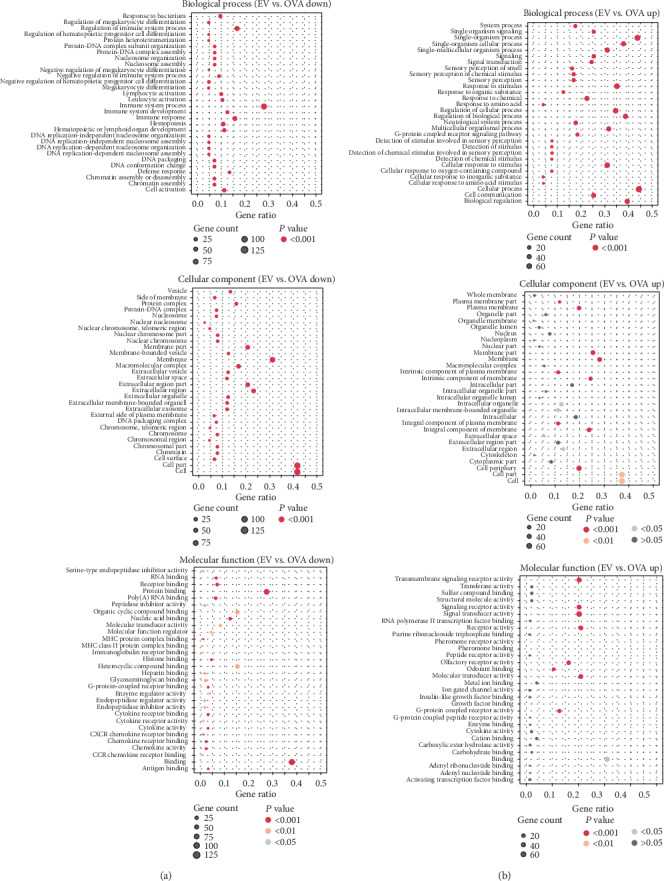
Bubble chart of gene ontology term association. Bubble size indicates the number of (a) downregulated or (b) upregulated genes for the corresponding annotation.

Up- and downregulated genes associated with each term were analyzed separately. DEGs that were downregulated following treatment with ASC-derived EVs were involved in whole-cell and within-cell components (Figure 6(a)). In contrast, genes differentially upregulated following treatment with ASC-derived EVs were strongly associated with cellular and single-organism processes, as well as biological regulation (Figure 6(b)).

### 3.6. KEGG Pathway Analysis

**Figure 7:**
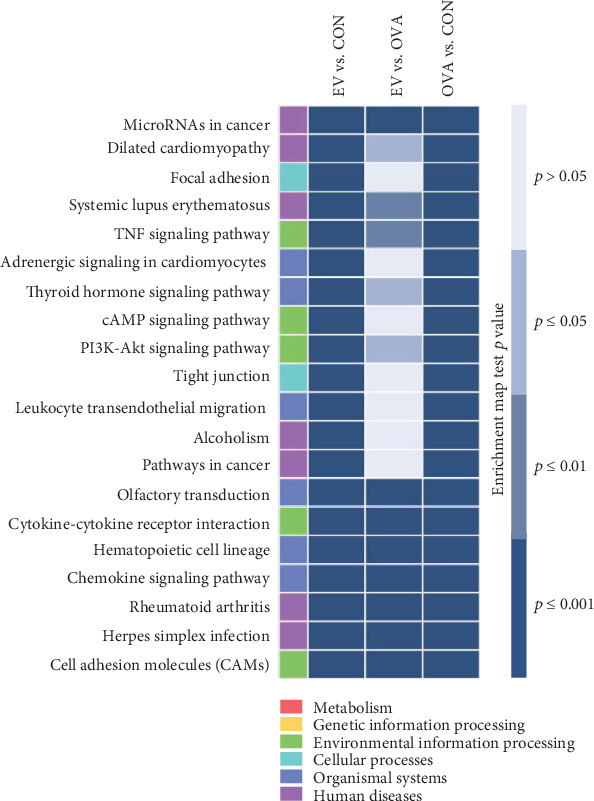
Enrichment analysis based on the Kyoto Encyclopedia of Genes and Genomes (KEGG) pathway identified genes with significantly differential expression. DEGs were strongly associated with environmental information processing, organismal systems, and human diseases. The expression of PON1, Bex2, Igfbp6, and Scgb1c1 was significantly increased following treatment with ASC-derived EVs in asthmatic mice. Gene ontology analysis showed that these upregulated genes were mainly involved in immune system processes and their regulation, cellular and single-organism processes, and biological regulation. These altered genes may be involved in the amelioration of allergic airway inflammation through treatment with ASC-derived EVs.

Enrichment analysis based on the KEGG pathway showed that highly significant DEGs following treatment with ASC-derived EVs were correlated with environmental information processing, organismal systems, and human diseases (Figure 7).

## 4. Discussion

MSCs have been reported as promising candidates for the treatment of allergic airway diseases [7–13]. However, MSCs have several drawbacks including immune rejection, risk of aneuploidy, difficulty of handling, and tumorigenicity. Previous studies have shown that ASC-derived secretome-containing EVs, even without ASCs, ameliorate allergic airway inflammation through the suppression of Th2 cytokine production and induction of Treg expansion [19, 22]. Furthermore, ASC-derived EVs have been shown to reduce static lung elastance and collagen fiber deposition in lung parenchyma and airways in experimental allergic asthma [29]. EVs exert their effects by delivering contents such as proteins, mRNAs, and microRNAs to recipient cells [30]. Recent studies have reported that mitochondrial transfer of MSCs, whose components can be found in EVs, can effectively alleviate allergic airway inflammation [31, 32]. The administration of MSC-derived EVs may reduce potential safety risks associated with stem cell therapy, suggesting that MSC-derived EVs may be a promising alternative to cell therapy for allergic airway diseases. However, the major pulmonary genes responsible for the immunomodulatory effects of MSC-derived EVs in allergic airway diseases have not been well documented.

Molecular and genetic research is required to elucidate the underlying immune suppression mechanism of MSC-derived EVs in Th2-mediated allergic airway inflammation. Microarray DNA hybridization techniques are widely applied in molecular biology research [33]. The DNA microarray consists of various DNA probes immobilized in groups on a solid support, forming an array of microspots [33]. When a DNA sample binds to the immobilized probe DNA thorough complementary sequence binding, detection is attained through reading the tagged markers attached to the target DNA [33]. The DNA microarray is a useful tool for the rapid, economical, and scalable identification of candidate DEGs associated with a phenotype [34]. The investigation of DEGs is essential for understanding and interpreting the immunomodulatory mechanism of MSC-derived EVs in allergic airway inflammation.

In this study, we performed DNA microarray analysis to identify DEGs associated with suppression of allergic airway inflammation by ASC-derived EVs. We performed hierarchical clustering of DEGs, followed by functional and pathway analyses. A total of 249 DEGs were identified, of which 228 and 21 were down- and upregulated, respectively, with a fold change of ∣log_FC_ | ≥2.0 between the EV and OVA groups. The genes Ear6, Ccl5, Ccl8, Ccl12, Tnfsf8, IL5Ra, and Tnfrsf13b were upregulated in the OVA group, but downregulated in the EV group. However, the genes PON1, Bex2, Igfbp6, Fpr1, and Scgb1c1 were downregulated by OVA sensitization and challenge, but upregulated by treatment with ASC-derived EVs. Genes downregulated after treatment with ASC-derived EVs were enriched in whole cells and cell components. However, those upregulated after treatment with ASC-derived EVs were strongly associated with cellular and single-organism processes and biological regulation. KEGG pathway analysis showed that DEGs following treatment with ASC-derived EVs were related to environmental information processing, organismal systems, and human diseases. In this study, we found that PON1, Bex2, Igfbp6, Fpr1, and Scgb1c1 expression decreased in lung tissues of asthmatic mice, but that PON1, Bex2, Igfbp6, and Scgb1c1 expression increased significantly following treatment with ASC-derived EVs. Together, these results suggest that PON1, Bex2, Igfbp6, and Scgb1c1 may be involved in the immune suppression mechanisms of ASC-derived EVs in allergic airway diseases.

PON1, a major antioxidant enzyme, has been reported to contribute to the pathogenesis of asthma [35] and many other diseases including rheumatoid arthritis [36, 37], diabetes [38], systemic lupus erythematosus [39], and psoriasis [40]. Recent studies have shown that PON1 expression and activity were significantly decreased in asthma and may have potential effects on asthma diagnosis [35, 41, 42]. Furthermore, PON1 decreased airway inflammation and airway remodeling in asthmatic mice and inhibited macrophage expression of LPS-induced inflammatory cytokines and lung fibroblast proliferation [43]. Bex2 regulates mitochondrial apoptosis and the G1 cell cycle in breast cancer [44]. A recent study demonstrated that Bex2 expression was suppressed by increased DNA methylation in IL-13-induced allergic airway inflammation [45]. Igfbp6 is an *O*-linked glycoprotein that has higher affinity to IGF-II than to IGF-I and is a specific inhibitor of IGF-II action [46]. Igfbp6 is also associated with cell growth and fibroblast proliferation in asthmatics [47]. Scgb1c1 is mainly expressed in the human respiratory tract mucosa and is downregulated by IFN-r and upregulated by IL-4 and IL-13 [48–50]. Scgb1c1 also plays an important role in protecting lung epithelial cells by recognizing and eliminating pathogenic microorganisms in the mucous membranes [48].

Our study had some limitations. Further evaluation of the effects of the genes identified in the present study on immunocytes such as T cells is required to clarify our findings. Future work should examine the specific functions of the identified DEGs in the suppression of allergic airway inflammation by ASC-derived EVs and investigate which components of ASC-derived EVs contributed to the regulation of these DEGs.

## 5. Conclusion

In this study, we revealed genetic information about the underlying immunomodulatory mechanism of ASC-derived EVs in allergic airways disease. We hypothesize that the identified genes (PON1, Bex2, Igfbp6, and Scgb1c1) lead to the amelioration of allergic airway inflammation, resulting in the improvement of allergic airway disease by ASC-derived EVs.

## Figures and Tables

**Table 1:** Genes downregulated following treatment with ASC-derived EVs.

Gene	OVA/CON	EV/CON	EV/OVA
Ear6	1.838022	1.149834	-1.598512
Ccl5	2.641300	1.198238	-2.204320
Ccl6	29.061333	16.230038	-1.790589
Ccl12	5.776364	3.477515	-1.661061
Tnfsf8	2.617759	1.697634	-1.542005
IL5Ra	2.587367	1.613224	-1.603849
Tnfrsf13b	1.985461	1.238539	-1.603067

ASCs: adipose stem cells; Ccl: chemokine ligand; CON: control; Ear6: eosinophil-associated ribonuclease A family member 6; EVs: extracellular vesicles; IL5Ra: interleukin-5 receptor alpha; OVA: ovalbumin; Tnfrsf13b: tumor necrosis factor receptor superfamily member 13B; Tnfsf8: tumor necrosis factor ligand superfamily member 8.

**Table 2:** Genes upregulated following treatment with ASC-derived EVs.

Gene	OVA/CON	EV/CON	EV/OVA
PON1	-9.35267	-6.482819	1.442686
Bex2	-3.798534	-2.489212	1.525998
Igfbp6	-3.230484	-2.120048	1.523779
Fpr1	-3.109904	-2.022899	1.537350
Scgb1c1	-2.224596	-1.467501	1.515908

ASCs: adipose stem cells; Bex2: brain-expressed X-linked 2; CON: control; EVs: extracellular vesicles; Fpr1: formyl peptide receptor 1; Igfbp6: insulin-like growth factor binding protein 6; OVA: ovalbumin; PON1: paraoxonase 1; Scgb1c1: secretoglobin family 1C member 1.

## Data Availability

The data used to support the findings of this study are available from the corresponding author upon request.
